# Intracranial internal carotid artery calcification is not predictive of future cognitive decline

**DOI:** 10.1186/s13195-022-00972-2

**Published:** 2022-02-11

**Authors:** Farzaneh Rahmani, Marina Nguyen, Charles D. Chen, Nicole McKay, Aylin Dincer, Nelly Joseph-Mathurin, Gengsheng Chen, Jingxia Liu, Hilary L. P. Orlowski, John C. Morris, Tammie L. S. Benzinger

**Affiliations:** 1 Mallinckrodt Institute of Radiology, Washington University School of Medicine, St. Louis, 510 South Kingshighway Boulevard, Campus Box 8131, St. Louis, MO 63110 USA; 2 Charles F. and Joanne Knight Alzheimer Disease Research Center (Knight ADRC), Washington University, St. Louis, MO USA; 3 Department of Neurology, Washington University in Saint Louis, St. Louis, MO USA; 4 Division of Public Health Sciences, Department of Surgery, Washington University School of Medicine (WUSM), St. Louis, MO USA

**Keywords:** Internal carotid artery, Calcification, Clinical Dementia Rating, White matter hyperintensities, Mini-Mental State Exam, 11C-Pittsburgh compound B, PiB, Centiloid

## Abstract

**Background:**

Intracranial internal carotid artery (ICA) calcification is a common incidental finding in non-contrast head CT. We evaluated the predictive value of ICAC (ICAC) for future risk of cognitive decline and compared the results with conventional imaging biomarkers of dementia.

**Methods:**

In a retrospective observational cohort, we included 230 participants with a PET-CT scan within 18 months of a baseline clinical assessment and longitudinal imaging assessments. Intracranial ICAC was quantified on baseline CT scans using the Agatson calcium score, and the association between baseline ICA calcium scores and the risk of conversion from a CDR of zero in baseline to a persistent CDR > 0 at any follow-up visit, as well as longitudinal changes in cognitive scores, were evaluated through linear and mixed regression models. We also evaluated the association of conventional imaging biomarkers of dementia with longitudinal changes in cognitive scores and a potential indirect effect of ICAC on cognition through these biomarkers.

**Results:**

Baseline ICA calcium score could not distinguish participants who converted to CDR > 0. ICA calcium score was also unable to predict longitudinal changes in cognitive scores, imaging biomarkers of small vessel disease such as white matter hyperintensities (WMH) volume, or AD such as hippocampal volume, AD cortical signature thickness, and amyloid burden. Severity of intracranial ICAC increased with age and in men. Higher WMH volume and amyloid burden as well as lower hippocampal volume and AD cortical signature thickness at baseline predicted lower Mini-Mental State Exam scores at longitudinal follow-up. Baseline ICAC was indirectly associated with longitudinal cognitive decline, fully mediated through WMH volume.

**Conclusions:**

In elderly and preclinical AD populations, atherosclerosis of large intracranial vessels as demonstrated through ICAC is not directly associated with a future risk of cognitive impairment, or progression of imaging biomarkers of AD or small vessel disease.

## Introduction

Mineralization of the intimal layer of the vessel wall is an integral part of the atherosclerotic process [1]. Calcification of the cervical internal carotid artery (ICA) is a well-studied example that is associated with the presence of cardiovascular risk factors and risk of stroke [2].

Intracranial ICAC (ICAC) is an expression of intracranial atherosclerosis and a common incidental finding on non-contrast computed tomography of the head (CT) [3]. Its prevalence ranges from 46 to 82% in the general adult population to almost 100% in individuals older than 90 years [4]. The most common sites of calcification are the cavernous carotid and the carotid siphon where the severity of calcification is associated with the presence of small vessel disease and white matter lesions [5–8], both of which have been shown to adversely affect cognition in older adults [9].

Studies have demonstrated an inverse relationship between the severity of intracranial ICAC and cognitive performance in terms of memory, executive function, global cognition, and processing speed in healthy adults [10, 11], as well as a relationship between extracranial ICAC and the risk of dementia [12]. Disrupted cerebral blood flow autoregulation, blood-brain barrier dysfunction, and increased amyloid deposition following increased ICA stiffness are among the suggested underlying mechanisms [13]. Little is known about the association of intracranial ICAC and Alzheimer disease (AD), in particular its association with imaging biomarkers of AD, including β-amyloid deposition and cortical and hippocampal atrophy.

We conducted a retrospective cohort study on 230 participants to assess the relationship between intracranial ICAC and imaging biomarkers of AD and small vessel disease as well as cognitive outcomes. The primary objectives of the study were to investigate (1) whether there is a relationship between the presence and severity of baseline ICAC and risk of conversion from normal to impaired cognition and (2) whether ICAC can predict future cognitive outcomes through longitudinal analyses. As a secondary objective, we investigated (3) whether there is any association between baseline ICAC and longitudinal changes in imaging biomarkers of AD and small vessel disease and (4) whether there is any association between imaging biomarkers of AD and small vessel disease and longitudinal changes in cognitive scores. We used the latter results to further investigate any potential indirect effect of ICAC on cognition mediated through its effect on these imaging biomarkers.

## Methods

### Participants

**Fig. 1:**
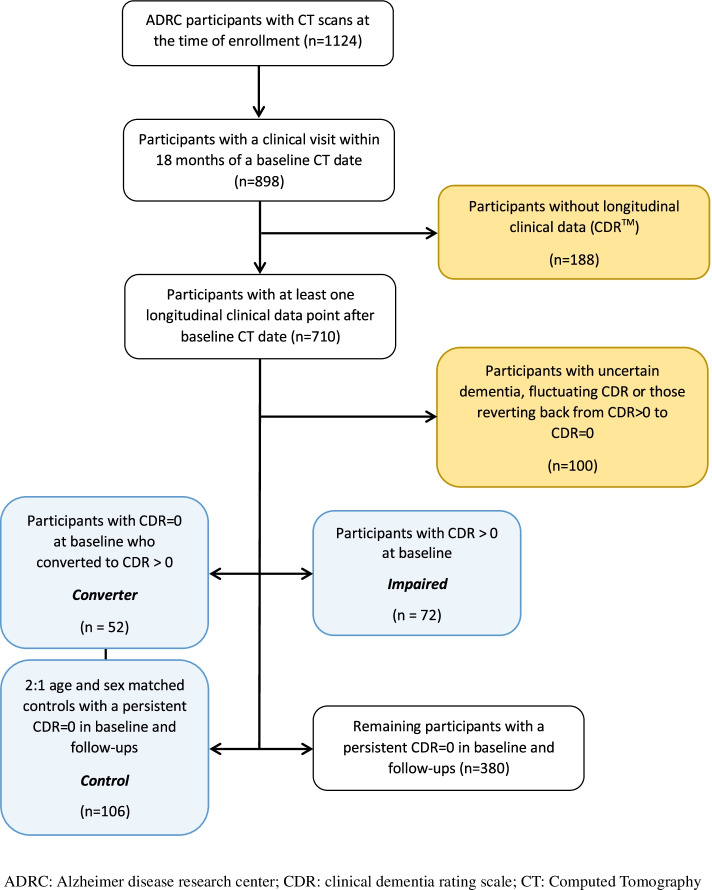
Inclusion criteria for the study population and reasons for exclusion

Participants were selected from a cohort of individuals enrolled and recruited from February 2009 through March 2018 in the ongoing longitudinal studies of memory and aging at the Charles F. and Joanne Knight Alzheimer Disease Research Center (Knight ADRC) at the Washington University School of Medicine in St. Louis. Inclusion criteria for this study were (1) having a PET-CT scan within 18 months of a clinical assessment, which was considered the baseline visit, and (2) having at least one follow-up clinical assessment. Among these, 52 participants were identified to convert from a Clinical Dementia Rating™ scale (CDR™) [14] equal to 0 in their baseline visit to a CDR > 0 in any of their follow-up assessments (case group or *converters*). A 2:1 age- and sex-matched *control* group was selected from among participants who had a CDR = 0 at baseline and remained cognitively normal (CDR = 0) in all follow-up assessments (*n* = 106). Finally, an *impaired* group was defined based on all participants who had a CDR > 0 at their baseline visit (*n* = 72). Figure 1 provides a summary of inclusion and exclusion criteria and how the final sample size was arrived at. To summarize, a total number of 230 participants (age range 52–90 years) were enrolled as part of the *control* (*n* = 106), *converter* (*n* = 52), and *impaired* (*n* = 72) groups. Exclusion criteria included (1) reverting back from CDR > 0 to a CDR = 0 at any follow-up visit, (2) diagnosis of uncertain dementia, and (3) fluctuating between CDR > 0 and CDR = 0 at any time during follow-up visits. Figure 1 gives an overview on how participants in each group were selected and the number of participants excluded due to each reason.

Within the impaired group, 62 participants had a diagnosis of AD dementia defined based on a comprehensive clinical assessment including a detailed interview of a collateral source, a neurologic examination by a skilled physician, and the CDR score [15]. Also, 10 participants from the impaired group had AD dementia with co-morbid active major depressive disorder. None of the participants demonstrated atypical variants of AD including the logopenic (primary progressive aphasia) or posterior cortical atrophy variants. Among participants who converted, 37 participants had a diagnosis of AD dementia followed by 9 participants who scored 0.5 only in the memory domain of CDR, 3 participants with AD dementia and co-morbid active major depressive disorder, one participant with primary Parkinson disease dementia, one participant with frontotemporal dementia, and one participant with vascular dementia.

### Clinical and psychiatric assessments

All participants underwent annual cognitive assessments which included a global CDR score evaluated by experienced clinicians utilizing a semi-structured participant interview and information from collateral sources. For longitudinal assessments of cognition, CDR Sum of Boxes score (CDR-SB), a summation of scores for each domain measured, was utilized [16]. Also included in the longitudinal cognitive assessment was the Mini-Mental State Examination (MMSE) score that was extracted from participants’ annual clinical visits. Finally, we derived the Preclinical Alzheimer Cognitive Composite (PACC) score for each individual as a measure of overall cognitive performance from a subset of the Knight ADRC cognitive battery as described before [17].

### Imaging assessments

#### CT scan calcium scoring

Non-contrast head CT scans were obtained using a Siemens Biograph 40 PET-CT scanner and transferred to a Vitrea 2 workstation (Vital Images Inc, Plymouth, MN). CT images were acquired using a tube voltage of 120 kV and tube current-time of 22 mAs in a 512 × 512 matrix. Scans all had 40 slices, included skull base to the vertex, had less than 0.6-mm isotropic spatial resolution, and slice thickness of 3 mm. Images were assessed for quality and absence of artifacts such as beam hardening or noise. Scans were reconstructed using the H19s algorithm and assessed using a bone window level (~1500 Hounsfield Units). A semi-automated coronary calcium scoring software (CT VScore^TM^, Cannon Medical Informatics) was used to calculate the Agatston calcium score and volume [18].

**Fig. 2:**
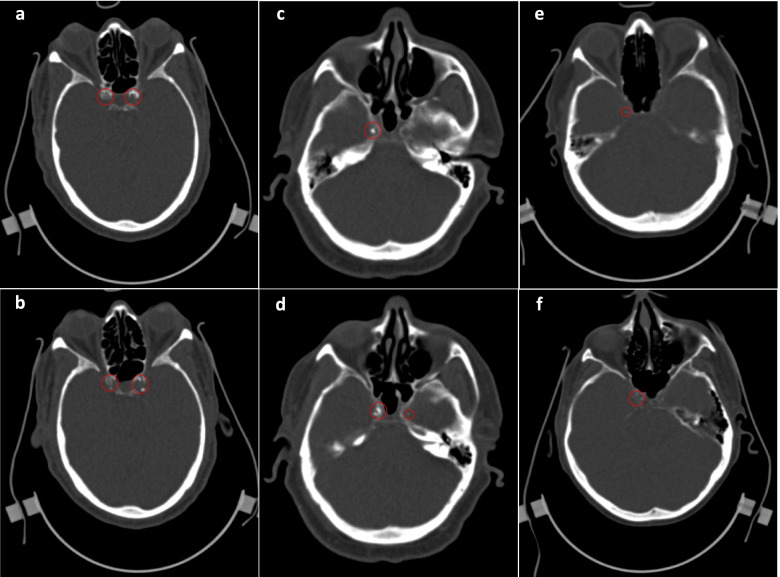
Internal carotid artery (ICA) calcification in low resolution CT scans for two participants. **a**, **b** Participant A demonstrates calcifications in the carotid siphon (total Agatston score: 1256). In participant B (**c**, **d**), calcifications are demonstrated in the petrous (**c**) and cavernous (**d**) segments of the left ICA (total Agatston score: 763). Mild ICA calcification is demonstrated in **e**–**f** in participant C with a total Agatston score of 150

The training cohort was defined by randomly choosing 15% of the 230 participants. Once trained to the satisfaction of the board-certified neuroradiologist rater (H.O.), two raters (M.N. and F.R.) scored the entire set blindly and independently by drawing regions of interest (ROIs) around areas of ICAC starting from the distal petrous apex to the ICA terminus (cavernous, clinoid, ophthalmic, and communicating segments). Figure 2 demonstrates examples of ICA calcification in the cavernous and clinoid segments for two participants. For this purpose, Agatston calcium scores were divided into four categories based on the cut-off values used to classify coronary artery calcification in clinical practice [19]. According to this rating, a calcium score of 0 is considered as the absence of coronary calcification, while scores ranging from 1 to 100 are rated as discrete, 101 to 400 as moderate, and scores above 400 as accentuated. The raters achieved a two-way agreement intraclass correlation coefficient (*ICC*) = 0.845 on raw calcium scores and an *ICC* = 0.889 on calcium score categories. Categorization of ICA Agatston scores based on cut-off values established for coronary artery calcium scoring was only performed to calculate the ICC between raters. These categories were therefore not used in any of the statistical analyses that followed. CT scans that received scores that differed between the two raters to the extent of receiving scores that belonged to different categories were blindly scored a second time, and any remaining discordant scans received a final resolution score by the board-certified experienced rater (H.O.). As both ICA calcium score and volume had a non-normal distribution, the natural log (Ln)-transformed values of both measures were used after adding one unit to the non-transformed values to deal with calcium scores of zero. Averaged scores between raters were used for analyses.

#### Amyloid PET acquisition and processing

Participants underwent amyloid PET imaging using either 11C-Pittsburgh compound B (PiB) or 18F-AV45 (florbetapir) radioligands. Methods for PiB and AV45 PET acquisition have been described previously [20, 21]. All scans were obtained on a Siemens Biograph 40 PET-CT or Biograph mMR and preprocessed using a unified longitudinal pipeline that outputs Centiloid values in order to standardize the PiB and AV45 tracers [22]. The amyloid PET data acquired on the day of the PET-CT scan was used to determine the amyloid status of all participants in their baseline. Amyloid positivity was defined as Centiloid values of > 16.4 in all visits [22].

#### MRI acquisition and processing

White matter hyperintensities (WMH) volume, total hippocampal volume, and AD cortical signature thickness measurements were calculated for all baseline and longitudinal MRI assessments using the unified processing pipelines described below. T1- and T2-weighted images were acquired using a magnetization-prepared rapid gradient-echo sequence on the Siemens 3T TIM Trio or Biograph mMR scanners. T1-weighted images were acquired with a 1 × 1 × 1 mm resolution, 2400 ms repetition time, 3.16 echo time, 8° flip angle, 176 frames, and a 256 × 256 field of view in sagittal orientation, and T2-weighted images were acquired with a 1 × 1 × 1 mm resolution, 3200 ms repetition time, echo time of 455, 120° flip angle, and a 256 × 256 field of view.

##### White matter hyperintensity volume

WMH volumes were calculated from a T2-weighted fluid-attenuated inversion recovery (FLAIR) and a T1 scan using the lesion segmentation toolbox (LST) implemented within SPM8 [23].

##### Total hippocampal volume and AD cortical signature thickness

Total hippocampal volumes were obtained on a T1-weighted image with the use of automated FreeSurfer 5.3. segmentation as previously described [24]. AD cortical signature thickness was also obtained from the T1-weighted image through an ROI cortical map representing the specific brain regions most susceptible to AD-related cortical atrophy as described previously [25].

### Statistical analysis

Statistical analyses were performed using IBM SPSS Statistics version 27 and R version 4.1.1. Normality of distribution of all variables was tested through the Kolmogorov-Smirnov goodness-of-fit test. Baseline variables were compared between the three cognitive groups (control, converter, and impaired) using the ANOVA or Kruskal-Wallis tests depending on the presence or absence of a normal distribution for each variable, respectively. Similarly, comparisons between participants with and without ICAC were conducted using the independent *t*-test or Mann-Whitney *U* test for variables with and without a normal distribution respectively. Correction for multiple comparisons using the false discovery rate (FDR) through the Benjamini-Hochberg method was performed when appropriate [26].

The binary logistic regression model was used to evaluate the association of baseline variables with the odds of conversion to CDR above 0 (odds of conversion ~ Ln-transformed ICA calcium score/volume, age, cognitive scores in baseline, biomarkers of AD, and small vessel disease in baseline) as well as the odds of ICAC (odds of the presence of ICAC ~ biomarkers of AD and small vessel disease in baseline, age). Univariable analyses were conducted first to identify variables with significant association with the outcome that were then entered into a multivariable analysis through stepwise selection [27]. A significance level of 0.3 was required to allow a predictor into the multivariable model and a significance level of 0.1 was required for a predictor to stay in the model where variables were selected through a backward likelihood ratio (LR) method in the multivariable model. Type I error probability of 0.05 was used to identify significant variables in all regression models. Survival analyses using the Cox proportional hazards (P-H) model were also used to investigate the effect of ICAC in different levels of baseline amyloid status with conversion to CDR > 0 as outcome (odds of conversion ~ ICA calcification*amyloid status). The P-H assumption was tested through adding a time-dependent variable to the model.

For longitudinal analyses, the R packages “lmerTest” and “lme4” were used to run a linear mixed model to extract the estimated annual rate of change in cognitive scores, WMH volume, and AD imaging biomarkers in the whole cohort including the control, converter, and impaired groups. Longitudinal measures of the variables of interest were considered dependent variables while time from baseline visit (*time*) and observations overtime for each subject (*time | subject*) were considered as fixed factors. Next, a simple linear model was devised to investigate the association between baseline Ln-transformed ICA calcium score and volume as an independent variable and estimated annual rates of change in cognitive scores and biomarkers of AD and small vessel disease (estimated annual rate of change in MMSE, PACC, CDR-SB, AD, and small vessel disease biomarkers ~ baseline Ln ICA calcium score/volume). A similar model was built to investigate the association between baseline biomarkers of AD and small vessel disease and estimated annual rate of change in MMSE and PACC score as dependent variables (estimated annual rate of change in MMSE and PACC score ~ biomarkers of AD and small vessel disease in baseline). Finally, the estimated marginal means of the dependent variables were plotted against time across different levels of the fixed effect of interest. Type I error probability of 0.05 was used to identify significant variables in all regression models.

Regression models described above allowed us to investigate the potential association of ICA calcium score/volume and imaging biomarkers of dementia and small vessel disease with longitudinal changes in cognitive scores. As a final step, we investigated any potential indirect effect of ICAC on longitudinal changes in cognition that was mediated through its effect on other relevant biomarkers. We used the R package “mediation” and implemented a design-based approach considering ICA calcium score or volume as exposure and longitudinal cognitive scores as outcome [28]. The annual rate of change in MMSE score was estimated for each participant using the method described for the longitudinal analyses and was considered as a dependent variable. The Ln-transformed ICA calcium score was considered an independent variable and WMH volume, hippocampal volume, AD cortical signature thickness, and Centiloid values were adopted as mediators in the model one at a time. The significance of each mediation model was tested using 1000 bootstrapped samples and considering a type I error probability of 0.05 as a significance level.

## Results

### No difference in ICA calcification, WMH volume, or AD imaging biomarkers between converters and controls

Figure 1 demonstrates a flow diagram showing the initial number of potentially eligible participants; selection criteria for the control, converter, and impaired groups; and those excluded for different reasons. A description of demographic, clinical, and cognitive risk factors of different groups in the baseline is presented in Table 1. There was no statistically significant difference between the control and converter groups in baseline calcium scores or volumes, WMH volume, or AD imaging biomarkers. The impaired group however had a higher WMH volume and Centiloid values as well as lower AD cortical signature thickness and total hippocampal volume compared to the control and converter groups. The presence of ICA was not associated with any significant difference in cognitive scores or AD imaging biomarkers. Men had more severe ICAC compared to women (Table 2).

**Table 1:** Demographics, cognitive status, vascular risk factors, and AD imaging biomarkers of participant groups in the baseline

	Total (*n*=230)	Control (*n*=106)	Converter (*n*=52)	Impaired (*n*=72)	*p*-value^§§^
Age, years (mean ± SD^§^)	73.7±6.7	73.2±6.6	73.1±6.7	74.8±6.9	0.418
Sex					
Men (*n* (%))	121 (52.6)	54 (50.9)	26 (50)	41 (56.9)	0.669
Women (*n* (%))	109 (47.7)	52 (49.1)	26 (50)	31 (43.1)	
Race					
Caucasian (*n* (%))	197 (85.6)	93 (87.8)	43 (82.7)	61 (84.7)	0.466
African-American (*n* (%))	25 (10.9)	11 (10.4)	6 (11.5)	8 (11.2)	
Native American (*n* (%))	1 (<1)	0 (0)	1 (1.9)	0 (0)	
Unknown (*n* (%))	7 (3)	2 (1.8)	2 (3.9)	3 (4.1)	
MMSE score (median (Q1–Q3))^§^	29 (28–30)	29 (29–30)	29 (28–30)^###^	28 (26–29)^***^	**<0.001**
CDR-SB (median (min–max))	0 (1)	0 (0–0.5)	0 (0–0.5)^###^	1.5 (0.5–6)^***^	**<0.001**
PACC score (median (Q1–Q3))	0.04 (−0.21–0.25)	0.13 (−0.03–0.41)	0.084 (−0.15–0.23)^#^	−0.23 (−0.41–0.04)^***^	**<0.001**
WMH volume, mm^3^ (mean ± SD)	16,072±24,128	18,024±19,131	21,819±18,562	29,603±26,163^**^	**0.005**
Ln ICA Ca score (median (Q1–Q3))	3.2 (0–4.8)	3.4 (0.69–4.8)	2.9 (0–4.05)	3.2 (0–5.2)	0.349
Ln ICA Ca volume (median (Q1–Q3))	3.7 (0–4.9)	3.94 (1.55–4.96)	3.6 (0–4.4)	3.5 (0–5.2)	0.520
Total hippocampal volume, mm^3^ (mean ± SD)	7004±967.6	7397±793	6991±844^#^	6397±1008^***^	**<0.001**
AD cortical signature thickness, mm (mean ± SD)	2.5±0.15	2.5±0.12	2.5±0.13^#^	2.4±0.17^***^	**<0.001**
Centiloid (mean ± SD)	6.9 (69.9)	3.4±35.3	66.5±83.2	15.3±75.1^***^	**<0.001**
Amyloid positivity (negative/positive)	114/116	70/36	23/29	21/51	**<0.001**

Asterisks represent significant post hoc tests following the Kruskal-Wallis or ANOVA models: *<0.05, **<0.005, and ***<0.0001 represent post hoc models showing the significant difference from the control group while ^#^<0.05, ^##^<0.005, and ^###^<0.0001 show significant difference from the impaired group

*Abbreviations*: *Control*, participants with CDR = 0 throughout all visits, *Converter*: participants converting from CDR = 0 to CDR > 0 in any of the follow-up visits, *Impaired*, participants with CDR > 0 in the baseline visit, *MMSE*, Mini-Mental State Examination, *CDR*, Clinical Dementia Rating Scale, *CDR-SB*, Clinical Dementia Rating Scale Sum of Boxes, *PACC*, Preclinical Alzheimer Cognitive Composite, *WMH volume*, white matter hyperintensities volume, *AA*, African-American, *C*, Caucasian, *Nat*, Native American, *U*, unknown race, *Ln ICA Ca score/volume*, natural log-transformed internal carotid artery Agatston calcium score/volume, *AD cortical signature thickness*, cortical thickness in signature regions affected in Alzheimer disease (see Dincer et al. 2020), *Centiloid*, measure of global amyloid disposition based on the conversion of PIB or AV45 PET SUVRs to a standardized scale. Amyloid positivity was defined based on Centiloid values > 16.4

^§^Data is reported in form of mean ±standard deviation (SD) for normally distributed variables and as median plus the first and third quartiles (Q1–Q3) for variables without normal distribution. Normality was determined using the Kolmogorov-Smirnov goodness-of-fit test

^§§^Kruskal-Wallis test was used to compare ICA Ca score and volume, MMSE, and PACC scores between the three baseline groups. Comparison for all other variables was conducted using an ANOVA model. Bold values indicate the test with statistical significance considering a threshold of 0.05

**Table 2:** Demographics, cognitive status, and AD imaging biomarkers of participants with and without internal carotid artery calcification in the baseline

	Calcification absent (*n*=64)	Calcification present (*n*=166)	*p*-value^§§^
Control/converter/impaired (*n*)	25/15/24	81/37/48	0.356
Age, years (mean±SD)	74.4±6.8	74.9±4.2	0.862
Sex			
Men (*n* (%))	22 (34.3)	99 (59.6)	**0.001**
Women (*n* (%))	42 (65.7)	67 (40.4)	
Race			
Caucasian (*n* (%))	50 (78.1)	147 (88.6)	0.110
African-American (*n* (%))	12 (18.7)	13(7.8)	
Native American (*n* (%))	0 (0)	1 (0.6)	
Unknown (*n* (%))	2 (3.1)	5(3)	
MMSE (median (Q1–Q3))^§^	29 (27–30)	29 (28–30)	0.061
CDR-SB (median (IQR))	0 (0.5)	0(1)	0.38
PACC score (median (Q1–Q3))^§^	0.039 (−0.23–0.23)	0.041 (−0.16–0.27)	0.334
WMH volume, mm^3^ (mean±SD)	15841±14220)	24424±23447	0.173
Total hippocampal volume, mm^3^ (mean±SD)	7052±878	7203±890	0.727
AD cortical signature thickness, mm (mean±SD)	2.5±0.17	2.5±0.11	0.771
Centiloid (mean±SD)	15.3±73.4	5.3±75.9	0.502
Amyloid positivity (negative/positive)	30/34	84/82	0.660

Asterisks represent significant post hoc tests following the Kruskal-Wallis or ANOVA models: *<0.05, **<0.005, and ***<0.0001 represent post hoc models showing a significant difference from the control group while ^#^<0.05, ^##^<0.005, and ^###^<0.0001 show significant difference from the impaired group

*Abbreviations*: *Control*, participants with CDR = 0 throughout all visits, *Converter*, participants converting from CDR = 0 to CDR > 0 in any of the follow-up visits, *Impaired*, participants with CDR > 0 in the baseline visit, *MMSE*, Mini-Mental State Examination, *CDR*, Clinical Dementia Rating Scale, *CDR-SB*, Clinical Dementia Rating Scale Sum of Boxes, *PACC*, Preclinical Alzheimer Cognitive Composite, *WMH volume*, white matter hyperintensities volume, *AA*, African-American, *C*, Caucasian, *Nat*, Native American, *U*, unknown race, *Ln ICA Ca score/volume*, natural log-transformed internal carotid artery Agatston calcium score/volume, *AD cortical signature thickness*, cortical thickness in signature regions affected in Alzheimer disease (see Dincer et al. 2020), *Centiloid*, measure of global amyloid disposition based on conversion of PIB or AV45 PET SUVRs to a standardized scale. Amyloid positivity was defined based on Centiloid values > 16.4

^§^Data is reported in form of mean ±standard deviation (SD) for normally distributed variables and as median plus the first and third quartiles (Q1–Q3) for variables without normal distribution. Normality was determined using the Kolmogorov-Smirnov goodness-of-fit test

^§§^Mann-Whitney *U* test was used to compare MMSE and PACC scores between the two groups. Comparison for all other variables was conducted using an independent samples *t*-test. Bold values indicate test with statistical significance considering a threshold of 0.05

### Amyloid burden, but not ICA calcification, is associated with future risk of cognitive decline

According to existing literature, we investigated the association of hippocampal volume [29], cortical thickness [30], and amyloid burden [31] with the risk of conversion to CDR > 0. Using univariable logistic regression models, lower hippocampal volume, and higher Centiloid values, but neither Ln-transformed ICA calcium score nor volume was found to be associated with higher odds of conversion to CDR above zero (Table 3). In the multivariable regression model, lower total hippocampal volume and higher Centiloid values were independently associated with higher odds of conversion to CDR above zero (Table 3) (overall model *R*-square: 0.245, *p*-value < 0.001).

**Table 3:** Binary logistic regression model to predict the odds of conversion to CDR above zero based on baseline biomarkers

	Odds of conversion from CDR = 0 to CDR > 0			
	Univariable		Multivariable	
	*p-value*	*OR (95% CI)*	*p-value* ^*§*^	*OR* ^b^ *(95% CI)*
Age (years)	0.996	1 (0.951–1.051)	**-**	**-**
Sex (male versus female)	0.911	0.963 (0.496–1.87)	**-**	**-**
Ln ICA calcium score	0.210	0.891 (0.743–1.068)	**-**	**-**
Ln ICA calcium volume	0.265	0.899 (0.845–1.084)	**-**	**-**
WMH volum^a^ (1000 mm^3^)	0.340	1.01 (0.989–1.032)	**-**	**-**
Total hippocampal volume^a^ (100 mm^3^)	**0.006**	**0.940 (0.9**–**0.982)**	**0.071**	**0.958 (0.915**–**1.004)**
AD cortical signature thickness (mm)	0.125	0.110 (0.007–1.837)	**-**	**-**
Centiloid^a^ (5 unit)	**0.003**	**1.08 (1.027**–**1.136)**	**0.012**	**1.070 (1.015**–**1.127)**

*Abbreviations*: *ICA*, internal carotid artery, *WMH volume*, white matter hyperintensities volume, *Centiloid*, measure of global amyloid disposition based on conversion of PIB or AV45 PET SUVRs to a standardized scale, *AD cortical signature thickness*, cortical thickness in signature regions affected in Alzheimer disease (see Dincer et al. 2020), *Ln ICA calcium score/volume*, natural log transformation of internal carotid artery Agatston calcium score/volume, *OR (95% CI)*, odds ratio and 95% confidence interval

^a^Odds ratios are demonstrated for 1000-mm^3^ increments in WMH volume, 100-mm^3^ increments in total hippocampal volume, and 5-unit increments in Centiloid scale

^b^Significant variables from the univariable model were entered into a stepwise multivariable model where a probability of 0.3 was set to enter the variables into the model and a probability of 0.1 to remove variables from the model

^§^ *p*-values were corrected for multiple comparisons through estimation of the false discovery rate via the Benjamini and Hochberg method. Variables with a *p*-value of 0.05 were considered statistically significant in both regression models (denoted in bold)

### Severity of white matter disease and increased age predict ICAC at baseline

As neither Ln ICA calcium score nor Ln ICA calcium volume had a normal distribution, we investigated the odd of the presence of ICAC—using a cut-off of 0—or odds of high versus low ICA calcium score—using the median value for Ln ICA calcium score/volume as cut-off—as dependent variables in our regression model. Predictors were selected according to literature and included age and sex [32], AD imaging biomarkers [33], and imaging biomarkers of small vessel disease [6]. Increased age, male sex, and decreased AD cortical signature thickness were associated with the presence and severity of ICAC, and lower AD cortical thickness, and higher WMH volume were associated with the ICAC severity (Table 4). In the multivariable regression model, age was the only independent predictor of the presence of ICAC (Table 4).

**Table 4:** Linear regression model to explore baseline variables with statistically significant associations with ICA Calcification in baseline

	**Presence vs. absence of ICA calcification** ^**§§**^			
	Univariable		Multivariable	
	*p-value* ^***§***^	*OR (95% CI)*	*p-value* ^***§***^	*OR (95% CI)* ^b^
Age (years)	**<0.001**	**1.136 (1.08–1.195)**	**<0.001**	**1.11 (1.05–1.175)**
Sex (male versus female)	**<0.001**	**2.82 (1.54–5.14)**	0.078	1.9 (1.03–2.8)
WMH volume (1000 mm^3^)^a^	0.058	1.025 (1.002**–**1.049)	**-**	**-**
Total hippocampal volume (100 mm^3^)^a^	0.489	0.989 (0.958**–**1.02)	-	-
AD cortical signature thickness (mm)	**0.022**	**0.053 (0.006–0.480)**	0.582	0.499 (0.042**–**5.92)
Centiloid (5 units)^a^	0.49	0.985 (0.952**–**1.02)	-	**-**
	**High vs. low ICA calcification** ^**§§**^			
	Univariable		Multivariable	
	*p-value* ^***§***^	*OR (95%CI)*	*p-value* ^***§***^	*OR (95%CI)* ^b^
Age (years)	**<0.001**	**1.11 (1.06–1.164)**	**0.048**	**1.085 (1.015–1.159)**
Sex (male versus female)	**0.005**	**2.4 (1.40–4.16)**	0.168	1.6 (0.807**–**3.42)
WMH volume (1000 mm^3^)^a^	**0.005**	**1.030 (1.011–1.048)**	0.163	1.012 (0.991**–**1.033)
Total hippocampal volume (100 mm^3^)^a^	0.277	0.984 (0.956**–**1.012)	**-**	**-**
AD cortical signature thickness (mm)	**0.006**	**0.056 (0.008–0.397)**	0.262	0.964 (0.921**–**1.0080)
Centiloid (5 units)^a^	0.277	0.982 (0.950**–**1.015)	**-**	**-**

*Abbreviations*: *ICA*, internal carotid artery, *WMH volume*, white matter hyperintensities volume, *Centiloid*, measure of global amyloid disposition based on conversion of PIB or AV45 PET SUVRs to a standardized scale, *AD cortical signature thickness*, cortical thickness in signature regions affected in Alzheimer disease (see Dincer et al. 2020), *OR (95% CI)*, odds ratio and 95% confidence interval; the presence or absence of ICA calcification was determined based on natural log transformation of ICA calcium score/volume

^a^Coefficients are demonstrated for 1000-mm^3^ increments in WMH volume, 100-mm^3^ increments in total hippocampal volume, and 5-unit increments in Centiloid scale

^b^Significant variables from the univariable model were entered into a stepwise multivariable model where a probability of 0.3 was set to enter the variables into the model and a probability of 0.1 to remove variables from the model

^§^ *p*-values were corrected for multiple comparisons through estimation of the false discovery rate via the Benjamini and Hochberg method. Tests with a *p*-value of 0.05 were considered statistically significant in both regression models (denoted in bold).

^§§^Presence of ICA calcification was defined based on Agatston ICA calcium score/volume using a cut-off of 0. High versus low ICA calcification was determined using the median value for natural log transformation of ICA calcium score/volume as cut-off

Similar to the results from the binary logistic regression analyses described in the previous section, survival analyses using the P-H cox model revealed the absence of any association between ICAC in the baseline and risk of conversion (hazard ratio (*HR*) (95% *CI*): 0.866 (0.474–1.579), *p*-value *= 0.638*), while amyloid positivity (using a 16.4 cut-off for Centiloid values) was associated with a risk of conversion to CDR > 0 (*HR* (95% *CI*): 2.3(1.3–4.1), *p*-value = 0.04). There was no interaction between amyloid status and ICAC in predicting the conversion risk (*HR* (95% *CI*): 0.826 (0.242–2.817), *p*-value = 0.760).

### ICAC is not predictive of longitudinal changes in cognition, WMH volume, or AD imaging biomarkers

**Fig. 3:**
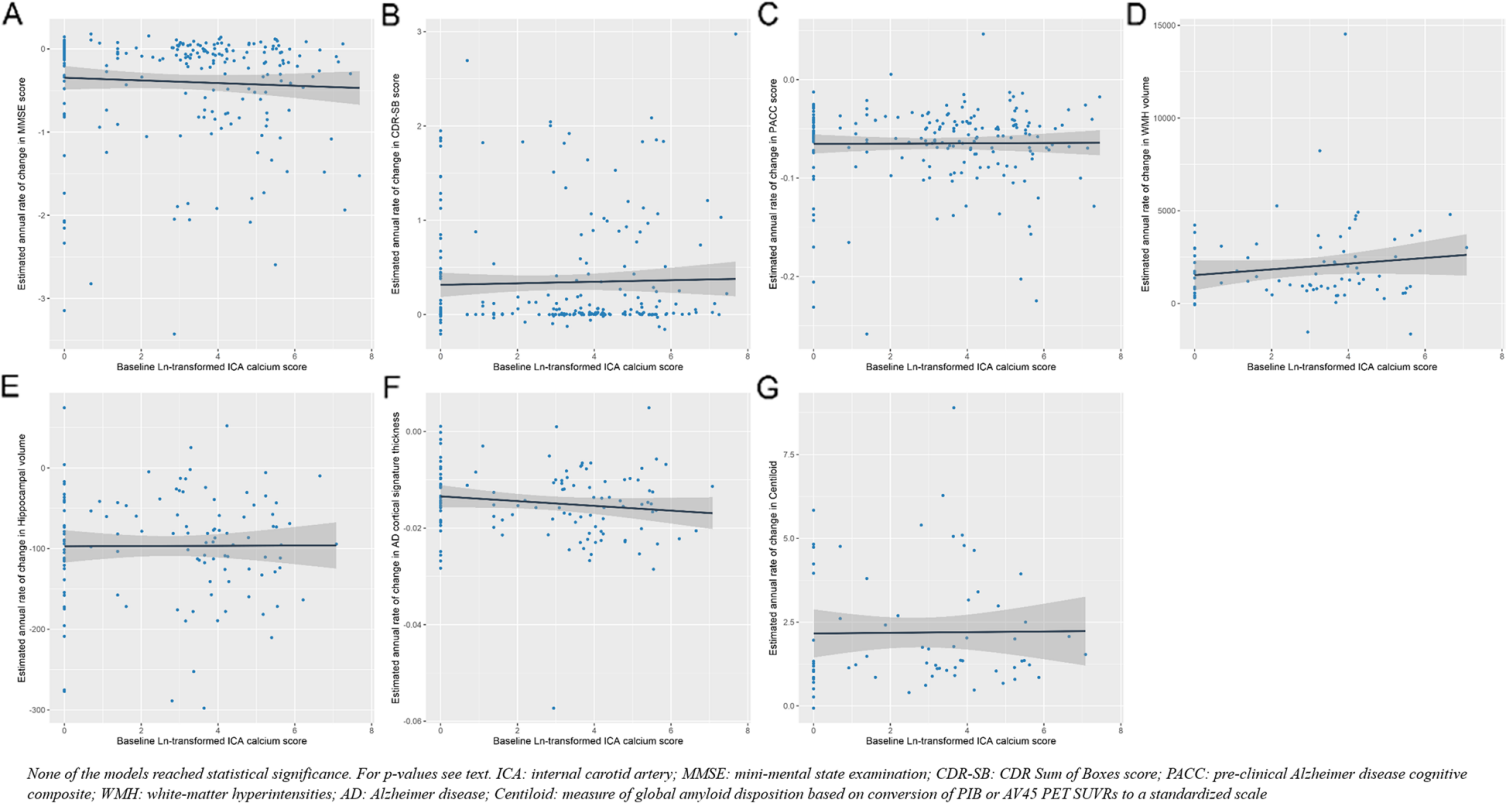
Association of the presence of internal carotid artery calcification in baseline with longitudinal cognitive scores and AD imaging biomarkers

Table 5 summarizes the number of patients and sessions used in each set of longitudinal biomarker analyses. Longitudinal analyses were all performed using the full cohort including the control, converter, and impaired groups. Univariable regression models were applied to evaluate the association of baseline variables with annual rates of change in cognitive scores and imaging biomarkers, estimated through a linear mixed model. Ln-transformed ICA calcium score or volume was not associated with any difference in the annual rate of change of MMSE, CDR-SB, or PACC score; WMH volume; total hippocampal volume; AD cortical signature thickness; or Centiloid values (Fig. 3 and Table 6). On the other hand, higher WMH volume and amyloid burden in the baseline as well as lower baseline hippocampal volume and AD cortical signature thickness were associated with a steeper rate of annual decline in MMSE and PACC scores (Table 7).

**Table 5:** Participants and session characteristics contributing to each set of longitudinal visits and variables

Visit type	Variables involved	Total included visits (*n*)	Number of participants (*n*)	Between visit gap, month (mean±SD)	Number of visits (median (lower–upper quartile))	Total person-time (months)
Clinical	CDR-SB/MMSE	1316/1257	230	14.5±6.7	3 (2–5)	9902
Psychometric	PACC score	1008	229	14.4±6.6	3 (2–5)	25018
MR session	AD cortical signature thickness/Total Hippocampal volume	341/341	118	25.5±18.3	2 (1–3)	14509
WMH session	WMH volume	214	83	30.6±16.8	2 (1–2)	5210
PIB/AV45 PET session	Centiloid	102	46	40.8±21.3	2 (1–2)	10699

*Abbreviations*: *MMSE*, Mini-Mental State Examination, *CDR*, Clinical Dementia Rating Scale, *CDR-SB*, Clinical Dementia Rating Scale Sum of Boxes, *PACC*, Preclinical Alzheimer Cognitive Composite, *WMH*, white matter hyperintensities, *PIB*, Pittsburgh compound B, *AV45*, 18F-AV45 (florbetapir), *Centiloid*, measure of global amyloid disposition based on the conversion of PIB or AV45 PET total cortical standardized uptake ratios to a standardized scale with arbitrary units ranging from 0 to 100, *Total Hipp*, total hippocampal volume, *AD cortical signature thickness*, cortical thickness in signature regions affected in Alzheimer disease

**Table 6:** Univariable linear regression model to explore the association of estimated annual rate of change of cognitive and imaging biomarkers based on baseline ICA calcium score and volume

Ln ICA calcium score as independent variable		
*Estimated annual rate of change in*	*p-value* ^*§*^	*Coefficient (SE)*
MMSE score	0.957	−0.016 (0.019)
CDR-SB score	0.957	0.008 (0.018)
PACC score	0.957	1.65e−4 (0.001)
WMH volume (mm^3^)	0.689	154.2 (118.5)
Total hippocampal volume (mm^3^)	0.957	0.161 (2.9)
AD cortical signature thickness (mm)	0.689	−4.39e−4 (3.41e−4)
Centiloid	0.957	0.01 (0.106)
Ln ICA calcium volume as independent variable		
*Estimated annual rate of change in:*	*p-value* ^*§*^	*Coefficient (SE)*
MMSE score	0.845	−0.015 (0.02)
CDR-SB score	0.845	0.007 (0.018)
PACC score	0.845	4.16e−4 (1.327e−3)
WMH volume (mm^3^)	0.7	0.153 (0.118)
Total hippocampal volume (mm^3^)	0.845	0.962 (2.94)
AD cortical signature thickness (mm)	0.7	−4.39e−4 (3.41e−4)
Centiloid	0.845	0.004 (0.021)

*Abbreviations*: *MMSE*, Mini-Mental State Examination, *CDR*, Clinical Dementia Rating Scale, *CDR-SB*, Clinical Dementia Rating Scale Sum of Boxes, *PACC*, Preclinical Alzheimer Cognitive Composite, *WMH volume*, white matter hyperintensities volume, *Centiloid*, measure of global amyloid disposition based on conversion of PIB or AV45 PET SUVRs to a standardized scale, *AD cortical signature thickness*, cortical thickness in signature regions affected in Alzheimer disease (see Dincer et al. 2020), *Ln ICA calcium score/volume*, natural log transformation of internal carotid artery Agatston calcium score/volume, *SE*, standard error of beta coefficient

^§^ *p*-values were corrected for multiple comparisons through estimation of the false discovery rate via the Benjamini and Hochberg method. Tests with a *p*-value of 0.05 were considered statistically significant in both regression models (denoted in bold)

**Table 7:** Univariable linear regression model to explore the association of baseline imaging biomarkers on dementia with annual rates of change in cognitive scores

*Baseline imaging biomarkers of dementia*	**Estimated annual rate of change in the MMSE score**	
	*p-value* ^*§*^	*Coefficient (SE)*
WMH volume (1000 mm^3^)^a^	**<0.001**	**−0.011 (0.002)**
Total hippocampal volume (100 mm^3^)^a^	**<0.001**	**0.036 (0.004)**
AD cortical signature thickness (mm)	**<0.001**	**2.36 (0.257)**
Centiloid (5 unit)^a^	**<0.001**	**−0.040 (0.004)**
*Baseline imaging biomarkers of dementia*	**Estimated annual rate of change in the PACC score**	
	*p-value* ^*§*^	*Coefficient (SE)*
WMH volume (1000 mm^3^)^a^	0.169	−2.66e−4 (1.87e−4)
Total hippocampal volume (100 mm^3^)^a^	**<0.001**	**0.002 (0.0003)**
AD cortical signature thickness (mm)	**<0.001**	**0.099 (0.021)**
Centiloid (5 unit)^a^	**<0.001**	**−1.969e−3 (3.45e−4)**

*Abbreviations*: *MMSE*, Mini-Mental State Examination, *CDR*, Clinical Dementia Rating Scale, *PACC*, Preclinical Alzheimer Cognitive Composite, *WMH volume*, white matter hyperintensities volume, *Centiloid*, measure of global amyloid disposition based on conversion of PIB or AV45 PET SUVRs to a standardized scale, *AD cortical signature thickness*, cortical thickness in signature regions affected in Alzheimer disease (see Dincer et al. 2020), *SE*, standard error of beta coefficient

^a^Coefficients are demonstrated for 1000-mm^3^ increment in WMH volume, 100-mm^3^ increment in total hippocampal volume, and 5-unit increment in Centiloid scale

^§^ *p*-values were corrected for multiple comparisons through estimation of the false discovery rate via the Benjamini and Hochberg method. Tests with a *p*-value of 0.05 were considered statistically significant in both regression models (denoted in bold)

### Effect of baseline ICAC on cognitive decline is fully mediated by white matter disease

**Fig. 4:**
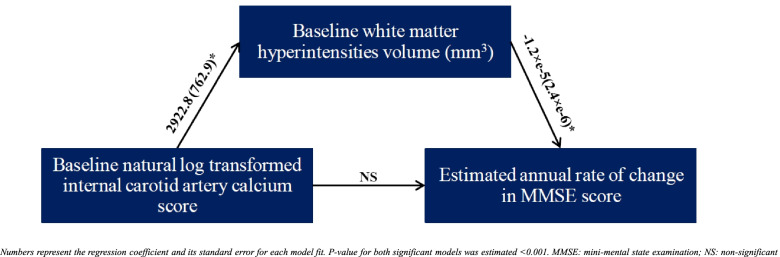
Mediation model between Ln-transformed ICA calcium score and the estimated annual rate of change in the MMSE score

Mediation analyses were used to investigate the presence of any potential indirect effect of ICAC on longitudinal cognition. Consistent with analyses described above, Ln-transformed ICA calcium score or volume was not significantly associated with longitudinal MMSE scores. However, mediation analyses revealed an indirect effect of baseline Ln-transformed ICA calcium score or volume on longitudinal MMSE, mediated solely by baseline WMH volume. As Fig. 4 illustrates, the regression model between baseline Ln-transformed ICA calcium score and WMH volume as well as the regression model between baseline WMH volume and estimated annual rate of change in MMSE was significant. The indirect effect of one unit increment in Ln-transformed ICA calcium score on the estimated annual rate of change in MMSE was therefore estimated to be −0.035 (95% *CI*: [−0.06; −0.02], *p*-value < 0.001). Similarly, the indirect effect of one unit increase in Ln-transformed ICA calcium volume on the estimated annual rate of change in MMSE was estimated as −0.034 (95% *CI*: [−0.05; −0.01], *p*-value < 0.001). In a similar set of analyses, total hippocampal volume, AD cortical signature thickness, and Centiloid values were unable to mediate any indirect effect between ICA calcium score or volume and longitudinal MMSE scores.

## Discussion

We conducted a retrospective cohort study on 230 adults from the Knight ADRC cohort to investigate the relationship between intracranial ICAC and cognitive outcomes, as well as imaging biomarkers of AD and small vessel disease. We demonstrated (1) an independent association between increased age and male sex with the odds and severity of ICA calcification; (2) no significant difference in the odds of conversion, longitudinal cognitive scores, WMH volume, hippocampal volume, AD cortical signature thickness, or amyloid burden between participants with and without ICAC in the baseline; (3) a significant indirect effect for baseline ICAC on longitudinal MMSE score which was purely mediated via baseline WMH volume; and (4) a significant association between higher WMH volume and amyloid burden as well as lower hippocampal volume and AD cortical signature thickness in predicting lower MMSE scores in longitudinal follow-ups.

Results obtained through analysis of participants’ baseline status were in line with previous literature indicating a direct relationship between age and severity of intracranial ICAC [2, 3]. Interestingly, although ICAC was not associated with increases in WMH volume during follow-up visits, it mediated longitudinal cognition through its association with baseline WMH volume. As WMH are primarily an expression of small vessel disease and ICAC is a proxy of large vessel atherosclerosis [34], our results indicate that while both findings reflect atherosclerosis—hence correlated in severity when looked in a cross-sectional manner—they represent different clinical trajectories of atherosclerosis in different intracranial vascular beds.

We identified no direct association between the presence or severity of ICAC and baseline or longitudinal cognitive scores (MMSE, CDR-SB, and PACC), nor did we find any association with the risk of conversion to CDR above 0. These results were in part in agreement with findings reported by Bos et al. from the Rotterdam study where one standard deviation increase in intracranial ICAC was able to predict the risk of dementia with borderline significance (*p*-value: 0.05) and odds ratio confidence interval of close to 1 (1.34 (1.01–1.78)) [12]. Here we show in a more rigorous manner and using a longitudinally followed cohort that intracranial ICAC was unable to predict conversion to CDR > 0 even in the setting of preclinical AD. Results from the same cohort as well as a cross-sectional report from the Thai population have identified a negative association between the severity of ICAC and longitudinal changes in general (i.e., MMSE) and domain-specific cognitive scores [10–12]. None of the above studies considered or controlled for the presence of concomitant white matter lesions as a confounding factor and potential proxy of vascular dementia. Further longitudinal studies are needed to understand the effects of ICAC on specific cognitive domains rather than global cognition measured through MMSE, CDR-SB, and PACC scores in the current study.

In vivo modeling of ICAC can be achieved through direct application of calcium chloride to arterial intima in rodents [35]. Rodents with ICAC demonstrate increased pulse pressure in distal, medium-sized arteries and a resulting impaired blood flow regulation in response to neuronal activity [13]. This would lead to increased blood-brain barrier permeability, amyloid deposition, and oxidative stress in the hippocampus, followed by increased cortical and hippocampal gliosis, culminating in neurodegeneration and memory impairment in these animals [13, 35, 36]. Similar to rodents with ICA calcification, Kang et al. identified an inverse relationship between the number of stenotic intracranial arteries and hippocampal volume, but no association with total cortical amyloid burden or AD cortical signature thickness [33]. Similarly, post-mortem AD brain assessments have shown associations between amyloid pathology and reduced hippocampal volume yet no such association is demonstrated between in vivo measurement of atherosclerosis and amyloid burden in patients with AD [37, 38]. When viewed in the context of animal model literature, our results present further evidence in human studies against the utility of ICAC in predicting in vivo AD imaging biomarkers in the setting of preclinical AD.

## Limitations

While calcifications of ICA are historically regarded as being a proxy of atherosclerotic disease and hence limited to the intimal layer, a growing body of literature suggest that calcification of intracranial ICA observed on CT is predominantly non-atherosclerotic and confined in the tunica media and internal elastic lamina of the blood vessels [3, 39]. Individuals with this medial ICAC are found to have different functional outcomes and more likely to benefit from endovascular treatment compared to those with intimal/atherosclerotic ICAC [40, 41]. Characterization of features in ICAC that would help identify the dominant type—intimal versus medial ICAC—requires a high-resolution CT scan with an optimal 0.6–1-mm slice thickness [42]. Nonetheless, the primary goal of this study was to use CT scans that have a quality similar to clinically available scans and use a tool that was available through the clinical workflow to investigate the significance of ICAC in predicting cognition. Therefore, non-contrast CT scans used for ICA calcium scoring in our study were obtained as part of the amyloid PET-CT scan performed during each participant’s enrollment (slice thickness: 3 mm and average 40 slices per image), creating a sub-optimal resolution for detection of features corresponding to each type of ICAC. On the other hand, the proximity of intracranial ICA to the petrous bone and bony skull base including the clinoid process limited our ability to delineate regions of interest around calcifications without including bone in the cavernous and clinoid segments of ICA. Differentiation between different ICAC subtypes and calcium scoring based on ICA segments were therefore beyond the sensitivity of the CT scans used in our study. Future studies should address this limitation through a prospective gathering of high-resolution CT scan data in the baseline and as part of follow-up imaging.

Importantly, the sample size pool for our longitudinal data points was different among different biomarker categories (Table 5), with the lowest number of data points and participants in the longitudinal Centiloid measures. We addressed this issue using linear mixed models to estimate the annual rates of longitudinal outcomes, a method known to be relatively unbiased in the presence of missing data [43].

Although ICA calcium score or volume did not show any predictive value for general cognitive outcomes in our longitudinal follow-up, cross-sectional data from the Rotterdam Study show evidence of an association between intracranial ICAC and worse performance in executive function, information processing speed, and motor speed domains [44]. These are among domains commonly associated with WM disease in the elderly population [45]. It is therefore imperative for future longitudinal studies to investigate the association of ICAC with changes in AD-specific cognitive domains. Last but not least, despite ongoing efforts in the Knight ADRC to enroll and more diverse and representative cohort of older adults, our study participants were primarily Caucasian.

## Conclusions

ICAC is frequently identified as an incidental finding on head CT scan and physicians are often uncertain about its prognostic and diagnostic value of such finding. Together, our results reveal that intracranial ICAC is unable to predict the onset of dementia in cognitively normal adults, nor it is able to predict longitudinal changes in cognitive scores, imaging makers of small vessel disease such as WMH volume, or AD imaging biomarkers. ICAC might however have an indirect effect on longitudinal cognition that is mediated by its effect on white matter disease. WMH volume might therefore be predictive of cognitive outcomes and hence an important incidental finding to report.

## Data Availability

De-identified participant data including clinical and cognitive assessment, CSF biomarkers, and dMRI images are available upon request through the Knight ADRC Leadership Committee (https://knightadrc.wustl.edu/research/resourcerequest.htm).
